# Dynamic Focusing (DF) Cone-Based Omnidirectional Fingertip Pressure Sensor with High Sensitivity in a Wide Pressure Range

**DOI:** 10.3390/s23208450

**Published:** 2023-10-13

**Authors:** Moo-Jung Seo, Jae-Chern Yoo

**Affiliations:** Department of Electrical and Computer Engineering, College of Information and Communication Engineering, Sungkyunkwan University, Suwon 440-746, Republic of Korea; mtothej92@skku.edu

**Keywords:** omnidirectional, dynamic focusing cone, pressure sensor, robot fingertip

## Abstract

It is essential to detect pressure from a robot’s fingertip in every direction to ensure efficient and secure grasping of objects with diverse shapes. Nevertheless, creating a simple-designed sensor that offers cost-effective and omnidirectional pressure sensing poses substantial difficulties. This is because it often requires more intricate mechanical solutions than when designing non-omnidirectional pressure sensors of robot fingertips. This paper introduces an innovative pressure sensor for fingertips. It utilizes a uniquely designed dynamic focusing cone to visually detect pressure with omnidirectional sensitivity. This approach enables cost-effective measurement of pressure from all sides of the fingertip. The experimental findings demonstrate the great potential of the newly introduced sensor. Its implementation is both straightforward and uncomplicated, offering high sensitivity (0.07 mm/N) in all directions and a broad pressure sensing range (up to 40 N) for robot fingertips.

## 1. Introduction

Dexterous robotic hands hold great promise as substitutes for humans in carrying out repetitive and hazardous tasks. To enable these robot hands to attain human-like dexterity when handling delicate or fragile objects, it is crucial to accurately and omnidirectionally sense the gripping pressure level exerted on an object so that one is able to ensure safe manipulation and prevent damage to the objects being handled [1].

Nonetheless, the task of achieving omnidirectional pressure sensing from a robot’s fingertip, while maintaining both high sensitivity and an extensive operational range, remains a challenge. Additionally, this capability must be attainable through a straightforward design and fabrication process [1,2,3,4].

Over the past ten years, there has been extensive research on advancing diverse pressure sensor technologies and transducer techniques. The majority of these approaches can be classified into four categories: piezoresistive [5,6,7,8], electromagnetic [9,10,11,12], micro-electro-mechanical [13,14,15,16], and capacitive [17,18,19,20,21,22,23,24,25,26].

The previously mentioned research endeavors primarily concentrate on enhancing sensitivity and broadening the range of detectable pressures. However, these studies do not prioritize achieving omnidirectional sensing.

As a result, these studies encounter numerous challenges when attempting to adapt their methods to fingertip pressure sensors that need to fulfill the combined criteria of excellent omnidirectionality, high sensitivity, and a suitably extensive pressure detection range.

For example, these approaches inherently require the attachment of multiple sensor arrays to the surface of each fingertip to accurately ascertain contact directions (positions). This leads to an escalation in both the quantity of electrical wires and the computational load necessary for processing a large amount of sensor information [27,28,29].

Lately, an increasing number of studies have aimed to address these challenges by introducing diverse vision-based sensors implemented on fingertip surfaces [30,31,32].

These vision sensors are utilized to predict the contact position, which is achieved by analyzing the deformation contours and patterns that emerge on the surface of a flexible finger.

K. Kamiyama and H. Kajimoto et al. [30] have developed a tactile sensor capable of measuring the distribution of force vectors on the sensor surface, which is accomplished by incorporating blue and red markers within its elastic body.

Upon the application of force onto the elastic body, the markers experience displacement. Subsequently, a CCD camera captures these marker displacements, aiding in the calculation of the distribution of force vectors.

Yuji Ito, Youngwoo Kim, and Goro Obinata [31] employed a CCD camera to record the sensor surface, which features irregularly arranged dots that shift when an external force is exerted on the fingertip. They were able to gauge a slippage degree on the fingertip surface by computing a stick ratio derived from the displacements of dots within the captured images.

In addition, an inventive optical tactile sensor [32] was introduced, showcasing super-resolution sensing capabilities. However, this design necessitates the construction of numerous miniature pins on the inner surface of a silicon membrane.

It employs an internally installed webcam to capture deformations on a pliable contact pad, subsequently computing the deformations in the sensor surface by assessing the displacements in molded internal pins.

In recent times, a newer and more improved development involves a fingertip sensor [33,34] that combines visual and tactile capabilities. This sensor features point markers positioned on the surface of the fingertip to enhance the precision of determining contact position and force. This enhancement is achieved by utilizing image-based data in conjunction with the deformation of each point marker caused by the application of contact force.

While these investigations effectively showcased the capabilities of vision-based pressure sensors, their performance might become unsatisfactory in scenarios involving fingertip surface contamination or visible scratches.

Additionally, when measuring a contact pressure with objects that share similar colors with the point markers, the vision camera can encounter difficulties in detecting deformations on these markers. This challenge can result in an inability to reliably estimate contact position and force.

In addition to these aforementioned sensors, there exists another category known as force-sensing resistor (FSR), which has been documented in the literature [35,36,37,38,39,40,41,42]. FSR sensors function in a way that their resistance diminishes as the force applied to the active sensing surface increases. FSR sensors are widely recognized for their exceptional sensitivity across a broad range of pressure levels. However, a notable drawback of FSR sensors lies in their limited precision.

In this current study, our objective is to introduce a novel fingertip pressure sensor referred to as the Dynamic Focusing Pressure (DFP) sensor for fingertips with the capability to visually detect pressure from all directions, thus offering omnidirectional sensitivity.

This sensor incorporates a 3D-printed dynamic focusing cone within the fingertip structure, ensuring visual pressure sensing. This innovative design enables the sensor to offer sensitivity from all directions, covering an extensive pressure detection range.

The DFP sensor is constructed with a hemispheric fingertip and a dynamic focusing (DF) cone, which are integrated into a 3D-printed plastic finger body, where the DF cone is positioned at the fingertip’s center and oriented vertically within the midpoint of the finger body’s hollow interior.

A miniaturized camera, featuring a fixed focal length, is also positioned directly above the DF cone. This arrangement allows the camera to capture an overhead view image of the cone.

As mechanical stress is applied vertically to the fingertip, the camera’s focal point shifts downward along the cone, coinciding with the cone’s upward movement towards the camera.

In this manner, as the applied pressure increases, the diameter of the cone observed in the camera image grows.

Consequently, the vertical pressure exerted on the fingertip surface can be readily determined by measuring the diameter of the cone in the top view image.

Although the introduced fingertip pressure sensor is quite simple and cost-effectively designed, which is an essential consideration for industrial applications, it offers remarkable sensitivity, an extensive detection range, and exceptional omnidirectionality. These qualities have been validated through experimental outcomes.

The rest of the paper is organized as follows:

In Section 2.1, the design and fabrication process of the proposed DFP sensor are explained. Section 2.2 provides a concise overview of the image-sensing mechanism employed by the pressure sensor. Moving on to Section 3.1, we conduct a comparative analysis of the pressure sensor’s performance in terms of sensitivity and working range with the existing previous works. Section 3.2 elaborates on how the proposed DFP sensor integrates a wall-shaped membrane to expand its elastic dynamic range.

Section 3.3 and Section 3.4 delve into the validation of the proposed DFP sensor’s superiority in terms of omnidirectionality and linearity. Lastly, in Section 4, we conclude by discussing the findings and outlining the path for future research endeavors.

## 2. Materials and Methods

### 2.1. Design and Fabrication of a Dynamic Focusing Pressure (DFP) Sensor

**Figure 1:**
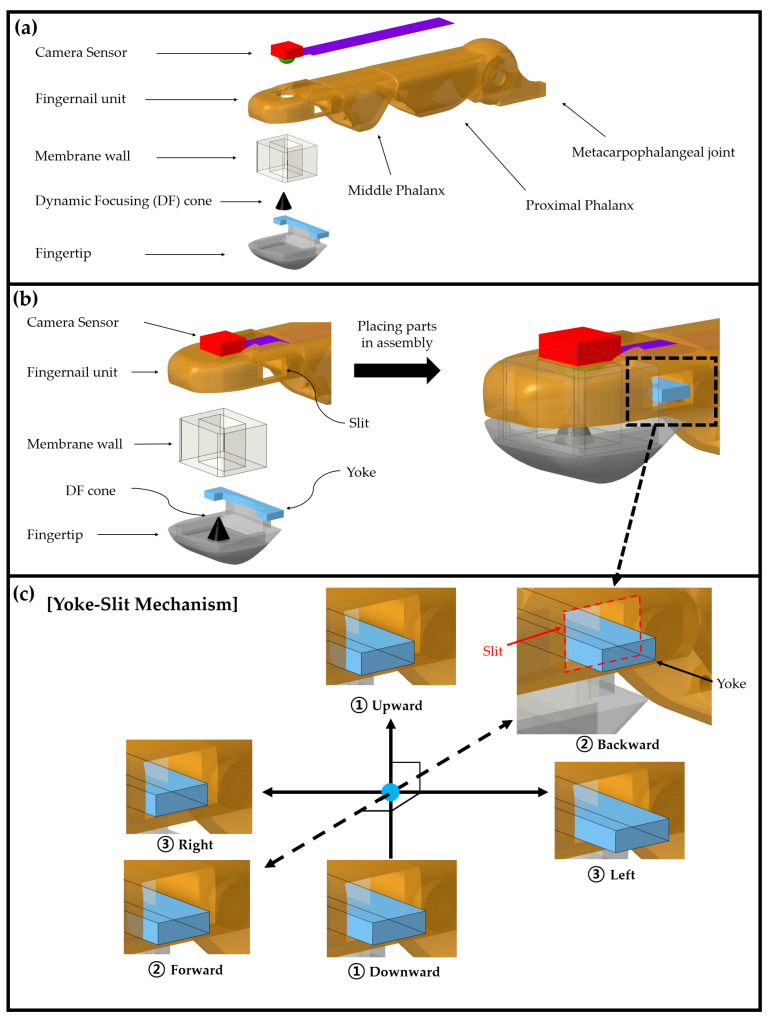
Simplified exploded view of the DFP sensor and its yoke-slit mechanism. (**a**,**b**) DFP sensor consists of a fingernail unit, fingertip, DF cone, omnidirectionally deformable membrane wall, and camera sensor. It also includes a built-in yoke-slit structure that physically binds the fingernail unit and the fingertip despite the fact that they are detached from each other. (**c**) Yoke inside a bound slit, which provides the fingertip with a DOF (Degree of Freedom) of movement with respect to the fingernail unit, thus the fingertip can freely move in all directions, but within the range defined by the slit.

**Figure 2:**
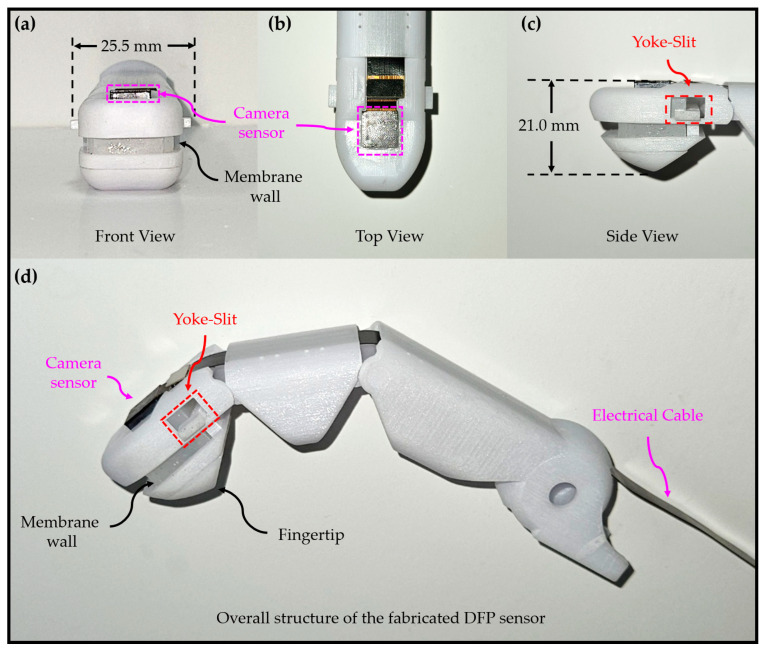
Images showing the fabricated DFP sensor. The DFP sensor, middle phalanx, proximal phalanx, and metacarpophalangeal joint are brought together in assembly to construct a robot’s index finger. (**a**) Front View. (**b**) Top View. (**c**) Side View. (**d**) Overall structure of the fabricated DFP sensor.

The omnidirectional-sensitive DFP sensor proposed in this study was designed using a 3D computer-aided design tool (Creo 8.0) to implement a pressure sensor that closely resembles the structure of a human fingertip, as depicted in Figure 1 and Figure 2.

The DFP sensor primarily consists of a 3D-printed fingernail unit and a fingertip, which were created using Projet MJP 2500 by 3D Systems Inc., based in Rock Hill, SC, USA. In this design, a DF cone is integrated on the top of the fingertip to constitute the complete structure of the finger body.

Crafted with dimensions of 3 mm in diameter and 3 mm in height, the DF cone was constructed using photosensitive resin material. This fabrication process employed the microArch P150 3D printer, which is known for its capability to achieve a printing resolution as fine as 25 µm.

To achieve real-time top-view imaging of the DF cone, an ultra-thin camera sensor (IMX219, ArduCAM, 3280 × 2544 pixels) was employed. This camera is a scant 5 mm thick but can still produce clear images while offering a broad viewing angle exceeding 62 degrees [43]. The camera is positioned in vertical alignment with the apex of the DF cone and seamlessly integrated through an aperture on the fingernail unit.

In this paper, the term “DF cone” represents the “dynamic focusing cone”. This nomenclature signifies that the camera has a fixed focal length but its focal point shifts dynamically along the axis of the cone. The shifts take place when the cone vertically moves in response to the pressure applied to the fingertip surface.

The DFP sensor has also a square-shaped membrane wall placed between the fingernail unit and the fingertip. This component serves to facilitate the cushionable movement of the fingertip and ensure a seamless return to its original position once the external mechanical stress is relieved.

The membrane wall exhibits human skin-like elastic properties [44,45,46], offering all directional flexibility.

As depicted in Figure 1c, a yoke-slit structure has also been incorporated into the finger body. This helps bind the fingertip to the fingernail unit and at the same time provides a limit for the maximum permissible vertical and horizontal displacement of the fingertip from the fingernail unit.

### 2.2. Pressure Sensing Mechanism of Using the DFP Sensor

Our DFP sensor operates through a vision-based sensing mechanism in which a compact camera is mounted via an opening on the fingernail unit. The camera captures the top view of the DF cone from a very close distance, enabling the measurement of the cone’s diameter in real time when pressure is applied.

**Figure 3:**
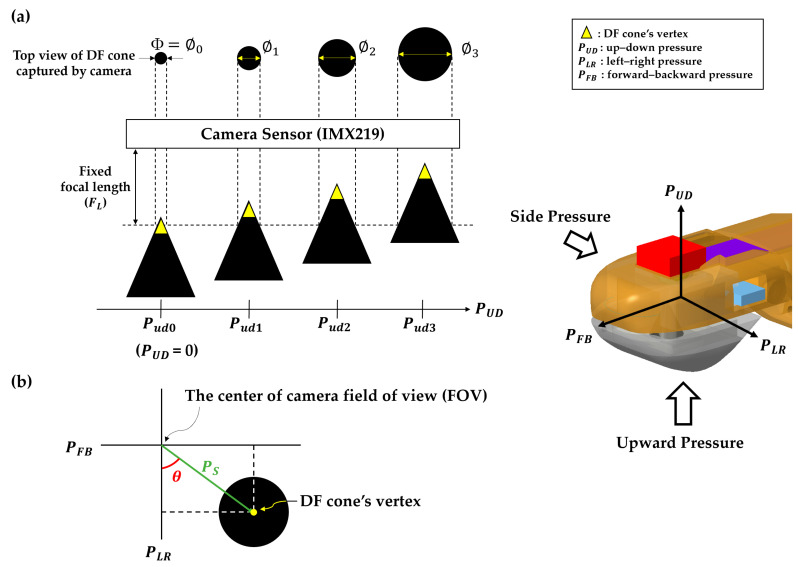
Pressure sensing mechanism based on the camera’s top-view image of the DF cone. (**a**) When an external force is vertically applied to the fingertip surface, the DF cone shifts upward towards the camera sensor. This movement results in an increase in the diameter of the cone captured by the camera. Here, Φ represents the diameter of the cone in the image captured by the camera, and $P_{UD}$ signifies the vertically applied pressure on the fingertip surface. (**b**) When there is lateral pressure to the fingertip, the vertex of the DF cone shifts away from the center of the camera’s field of view (FOV). The direction (*θ*) of the lateral pressure ($P_{S}$) can be determined through mathematical calculations by decomposing the vector $P_{S}$ into its $P_{FB}$ and $P_{LR}$ components on a Cartesian coordinate system, where the vector $P_{S}$ is the component formed by connecting the center of the camera’s FOV to the vertex of the DF cone.

As illustrated in Figure 3a, due to the camera sensor’s fixed focal length (F_L_: approximately 3 mm) and its consistent view of the cone’s top, the camera’s focal point shifts vertically downward along the cone’s axis when a vertically applied mechanical stress ($P_{UD}$) is exerted on the fingertip’s surface, resulting in increasing the camera-captured cone’s diameter (Φ).

For the purpose of explanation, let us denote the cone’s diameter under zero upward pressure ($i.e.,P_{UD}=0)$ as $\emptyset_{0}$. Initially, let us assume that the camera’s focal length is fixed at F_L_, letting it focus on the cone’s vertex as shown in Figure 3a. However, when upward pressure is applied to the fingertip ($i.e.,\;{\;P}_{UD}\neq 0)$, the cone moves upward, leading to a vertical downward shift of the camera’s focal point along the cone’s axis. Consequently, the diameter of the cone captured by the camera becomes greater than $\emptyset_{0}$.

As one would intuitively anticipate, the diameter Φ increases as the pressure increases. Consequently, the relationship of $\emptyset_{0}{<\emptyset }_{1}<\emptyset_{2}<\emptyset_{3}$ is obtained when upward pressure is applied to the fingertip with the condition of $P_{ud0}{<P}_{ud1}<P_{ud2}<P_{ud3}$.

Therefore, the vertical pressure applied to the fingertip surface can be easily computed by measuring the cone’s diameter from the top view image of the cone.

On the other hand, as depicted in Figure 3b, when an external force is applied to the side of the fingertip, the vertex of the DF cone shifts away from the center of the camera’s field of view (FOV), producing a side pressure component ($P_{S}$).

Here, let ${P_{UD},\;P}_{FB},$ and $P_{LR}$ represent the vertical (up–down), forward–backward, and left–right pressure components, respectively. It is important to note that these components are subject to be orthogonal to one another.

The side pressure component ($P_{S}$) can be decomposed into its $P_{FB}$ and $P_{LR}$ components along each respective coordinate axis, and then the direction (*θ*) of the side pressure ($P_{S}$) can be expressed using Equation (1).

(1) $$\begin{array}{c} P_{FB}=-P_{S}\sin\theta ,P_{LR}=P_{S}\cos\theta ,\;\mathrm{and}\\ \theta =-\tan^{-1}\frac{P_{FB}}{P_{LR}} \end{array}$$

Here, the following should be noted:

(i) In the top view captured by the camera, it is observed that the diameter Φ is at a minimum value when no vertical (up or down) pressure is applied.
(ii) The vertex of the DF cone aligns with the center of the camera’s field of view (FOV) in the absence of any lateral pressure applied to the fingertip surface.

Upon releasing the external force, the deformed membrane wall restores its original shape, facilitating the fingertip’s reversion to its initial position. This backward movement is effectively achieved through the elastic and tensile characteristics of the membrane wall.

**Figure 4:**
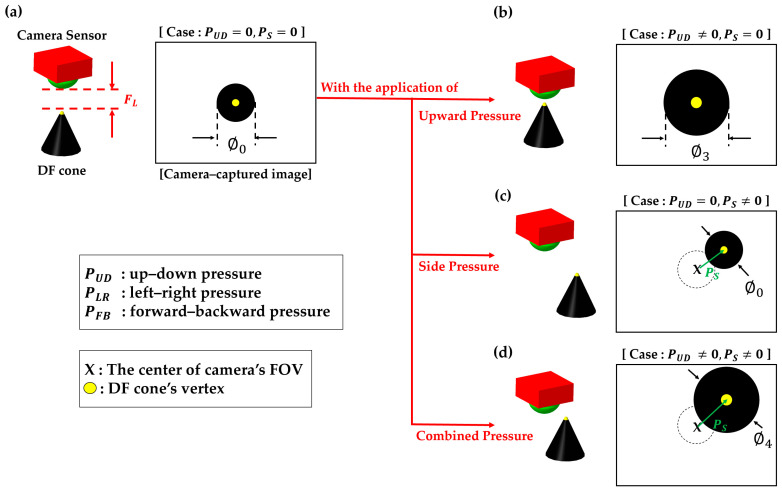
Schematic illustration of measuring the pressure applied to the fingertip surface from different directions. It uses the top-view image of the DF cone as captured by the camera sensor. Each panel depicts a distinct pressure application scenario representing (**a**) the initial state (*Case*: $P_{UD}=0\;and\;P_{S}=0)$, (**b**) an upward pressure (*Case*: $P_{UD}\neq 0\;and\;P_{S}=0)$, (**c**) a side pressure (*Case*: $P_{UD}=0\;and\;P_{S}\neq 0)$, and (**d**) a combined pressure (*Case*: $P_{UD}\neq 0\;and\;P_{S}\neq 0)$.

Figure 4 illustrates several representative examples demonstrating the methodology for measuring the pressure exerted on the fingertip surface using a camera-captured top-view image of the DF cone.

Figure 4a,b describes how the DF cone’s diameter changes with the application of vertical pressure, where it is seen that the camera-captured cone’s diameter increases as the pressure increases.

Figure 4a corresponds to a scenario with no applied pressure (${i.e.,\;P}_{UD}=0$ and $P_{S}$ = 0), while Figure 4b represents a situation where an upward pressure is exerted on the fingertip $({{i.e.,\;P}_{UD}\neq 0\;and\;P}_{S}=0$).

Therefore, the diameter ($\emptyset_{3}$) of the cone in Figure 4b becomes larger than its initial state (Φ = $\emptyset_{0})$, indicating that $\emptyset_{0}{<\emptyset }_{3}$.

Figure 4c,d explains how to determine the side pressure component ${(P}_{S})$ when there is a lateral pressure ${(P}_{S}\neq 0)$ against the fingertip surface, where ${\;P}_{S}$ is the vector that connects the center of camera’s FOV to the vertex of the DF cone.

Since Figure 4c corresponds to the case where there is no vertical pressure applied to the fingertip, the diameter of the cone remains at its initial state, i.e., Φ ${=\emptyset }_{0}$.

On the other hand, Figure 4d is when a combined pressure ${{(i.e.,\;P}_{UD}\neq 0\;and\;P}_{S}\neq 0$) is applied to the fingertip. In this case, it is expected that $\emptyset_{4}$ $>\emptyset_{0},$ along with non-zero values for both $P_{FB}\;and\;P_{LR}$.

## 3. Experimental Results and Discussion

### 3.1. Performance Comparison of Pressure Sensor in Terms of Sensitivity and Operational Range

**Figure 5:**
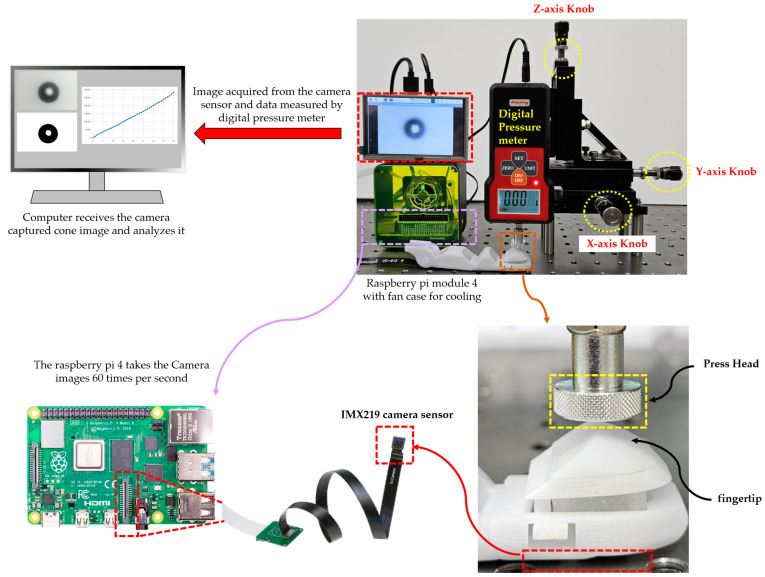
Measurement setup of the DFP sensor equipped with a high-precision digital pressure meter [47]. The high-precision digital pressure meter is mechanically linked and synchronized with a three-axis moving stage, ensuring that the press head of the digital pressure meter moves in tandem with the displacement adjustments from the xyz stage. The camera-captured image under illuminance of 420~450 lux and the corresponding data obtained from the digital pressure meter are analyzed to calculate the direction and magnitude of the pressure applied to the fingertip. This analysis involves the utilization of a MATLAB program version 2022b (MathWorks Inc., Natick, MA, USA) running on a personal computer.

The experiment was conducted on the measurement setup presented in Figure 5, which was designed to accurately measure the pressure applied to the fingertip, equipped with a high-precision digital pressure meter (Model SJ50N, Shenzhen, SanLiang, China), Raspberry Pi Model 4B (Raspberry Pi Foundation, London, UK) and a three-axis moving stage.

The Raspberry Pi facilitates the transfer of image data acquired from the camera sensor to a personal computer while adjusting the applied pressure strength and position by controlling the three-axis moving stage. As stated earlier, the focal length of the camera sensor is fixed and is initially configured to focus on the vertex of the cone that corresponds to when no external pressure is applied.

Subsequently, the diameter of the cone captured by the camera undergoes changes or the vertex of the DF cone shifts away from the center of the camera’s field of view, depending on the direction and strength of the pressure exerted on the fingertip.

For example, when the fingertip is pressed with a given force, the camera-captured cone’s diameter increases with increasing the vertically applied force.

Conversely, with an increase in lateral pressure exerted against the fingertip, the vertex of the DF cone moves farther away from the center of the camera’s field of view.

For comparative purposes, the performance of the proposed DFP sensor was evaluated concerning sensitivity and operational range in contrast to previous research studies.

The comparison results shown in Table 1 indicate a remarkable similarity between the performance of the DFP sensor and human skin. This is demonstrated by the DFP sensor’s omnidirectional sensitivity and its ability to encompass the entire spectrum of human touch pressure.

Even when benchmarked against the most advanced pressure sensing techniques available, the DFP sensor shows superior performance, particularly with regard to sensitivity and sensing range.

We achieved a sensitivity of 0.07 mm/N, coupled with a notably extensive working range of up to 40 N. Moreover, the straightforward fabrication process makes our sensor highly competitive compared to the sensors detailed in Table 1.

This superiority can be attributed to two main factors: Firstly, the DF cone induces a relatively substantial displacement (approximately ranging from 3 to 4 mm), resulting in enhanced sensitivity compared to other sensors with only a few micron displacements [20,24]. Secondly, the double-floored elastic membrane wall (as described in Section 3.2) offers an extended elastic deformation capacity, thereby expanding the working range of the sensor.

**Figure 6:**
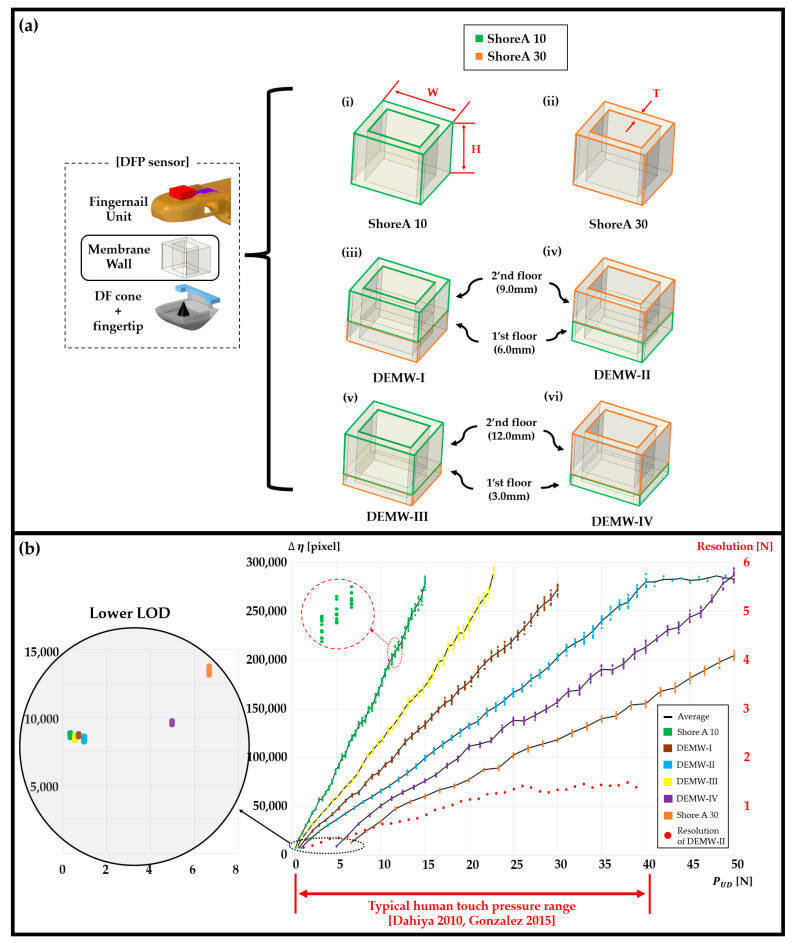
Comparative experiment of evaluating various membrane wall designs with different harness options, including (i) through (vi). The black solid lines indicate the average of Δη in each pressure level. (**a**) Several combinations involving variations in the height-to-height ratio between the 1st and 2nd floors, along with the choice of Shore A hardness for each floor of membrane wall. These combinations encompass both single-floored and double-floored membrane wall configurations. Each membrane wall has the size of 18.0 (W) × 15.0 (H) × 2.5 (T) (mm). (**b**) Plot of Δη versus pressure, with a tenth trial for each pressure level, where it is seen that DEMW-ⅠⅠ exhibits the widest operational range while keeping the smallest lower limit of detection [57,58].

In terms of pressure sensor resolution, the DFP sensor demonstrates a relatively impressive resolution of approximately 0.28 N on average for pressure levels below 10 N despite its significantly extensive working range (See Table 1 and Figure 6). Nevertheless, its resolution gradually diminishes as the pressure level increases. It is important to note that this reduction in resolution becomes less critical as the pressure level rises, as higher pressure levels often require less precision.

### 3.2. Double-Floored Elastic Membrane Wall (DEMW)

As depicted in Figure 6a, the proposed DFP sensor incorporates a wall-shaped membrane situated between the fingernail unit and the fingertip, providing highly elastic and recoverable properties, and thereby ensuring consistent and repeatable functionality as a pressure sensor.

When pressure is applied to the fingertip, the membrane wall undergoes deformation in the direction of the applied pressure. Conversely, upon releasing the pressure, the membrane wall returns to its initial state.

In this study, a novel specialized membrane wall was devised, referred to as the “double-floored elastic membrane wall” (DEMW), which serves to enhance the elastic dynamic range of the membrane wall.

The DEMW comprises two square-shaped elastomer membranes, each of which has a different hardness, which are stacked vertically and assembled. This novel design allows for greater elasticity and a wider dynamic range of deformation, depending on its optimization.

Elastomeric materials that are already available commercially offer a diverse range of hardness options, ranging from soft (20 Shore A) to firm (80 Shore A). This extensive variety provides the advantage of selecting materials tailored to specific applications.

For the purpose of imitating realistic human-like fingertip surfaces, each floor of the DEMW was crafted using silicone elastomeric materials with Shore A hardness of either 10 or 30, exhibiting skin-like properties in all directions [44,45,46].

To find the optimal condition that maximizes the elastic working range of the membrane wall, many experiments were exhaustively conducted while evaluating various combinations of height-to-height ratio between the 1st and 2nd floors, alongside the choice of Shore A hardness for the materials.

Figure 6a,b illustrates some representative combinations over different height-to-height ratios and Shore A hardnesses for selecting an optimal configuration of the membrane wall.

The outcome of the experiments revealed that the most optimal configuration was achieved with a height-to-height ratio of 3:2. Specifically, when combining a Shore A hardness of 30 for the 1st floor with a Shore A hardness of 10 for the 2nd floor, denoted as DEMW-II, the sensor demonstrated a robust linear relationship between pressure and Δη (see Section 3.3 for a detailed explanation of Δη). Additionally, this configuration showcased the widest working range under the condition of keeping a lower limit of detection (LOD) approaching zero (See Figure 6b).

It is evident that the working range of the DEMW-ⅠⅠ is notably broader and its LOD is also lower compared to that of other configurations of the membrane wall. This improvement is greatly attributed to the fact that the DEMW-ⅠⅠ is constructed by stacking two elastomer membranes, each possessing distinct hardness levels, thus being able to expand its elastic working range.

This is not surprising since the DEMW-II was designed with the intention of having the 1st floor softer than the 2nd floor, thereby resulting in a sequential compression pattern.

Initially, the 1st floor undergoes compression, followed by the subsequent compression of the 2nd floor as the applied fingertip pressure gradually increases.

As a result, we can ascertain that DEMW-ⅠⅠ is the best option in the sense that it not only offers the broadest elastic deformation range but also attains a near-zero lower limit of detection. Consequently, the DEMW-II stands out as the most suitable configuration of the membrane wall for achieving a human-like fingertip pressure sensor.

During this experiment, the thickness of the membrane wall was selected to facilitate easy removal from the mold without damaging thin and delicate parts while keeping enough elasticity to hold its shape. Meanwhile, the other dimensions were chosen while taking into account the need for a structure that closely resembles a human-like fingertip.

### 3.3. Experimental Analysis of the Omnidirectionality of DFP Sensor

**Figure 7:**
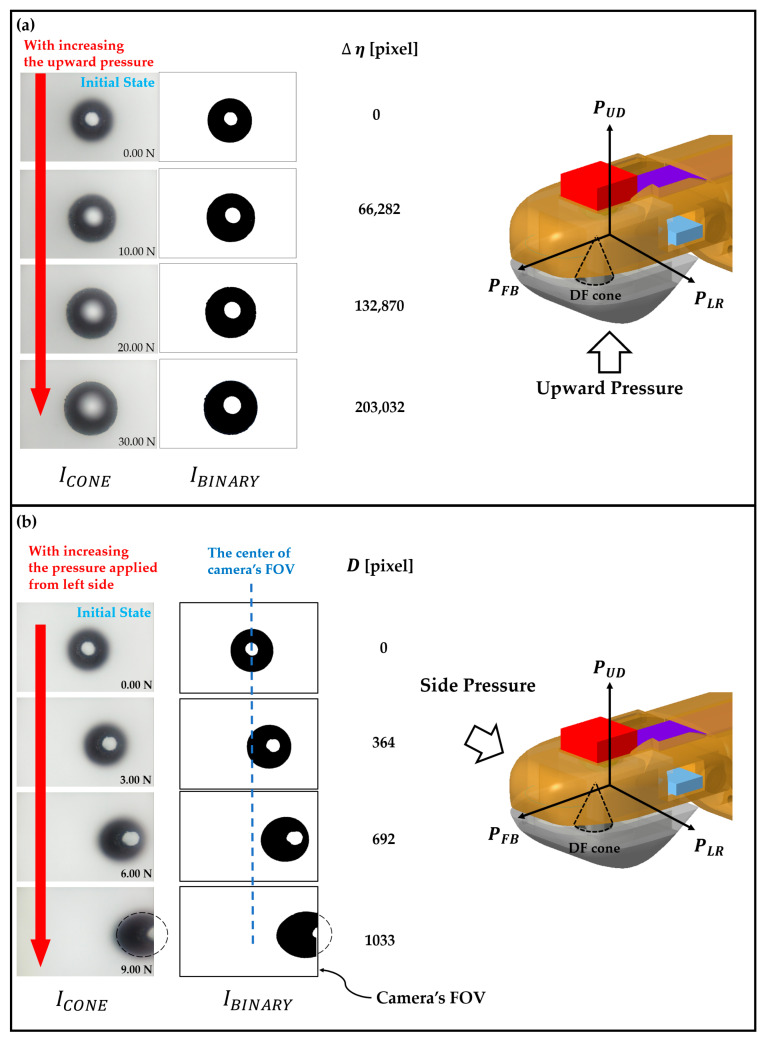
Camera-captured cone image ${(I}_{CONE})$ and its corresponding binary image ${(I}_{BINARY})$ according to the pressure applied to the fingertip surface. The changes in the binary image provide crucial information about the direction and magnitude of the applied pressure on the fingertip surface. (**a**) The delta black pixel ∆η increases as the upward pressure increases. It is important to note that in real experimental scenarios, there might be some minor side pressure components included with the upward pressure, which could slightly affect the measurements. This might be due to the difficulty of perfectly isolating purely vertical pressure without any lateral components. (**b**) The deviation distance ***D*** increases as the lateral pressure increases. When a side pressure is exerted on the fingertip, the vertex of the DF cone moves away from the center of the camera’s field of view (FOV). It is seen here that the circular shape of the cone appears slightly distorted in the captured image due to the oblique camera angle, which is expected to be more skewed with more side pressure.

**Figure 8:**
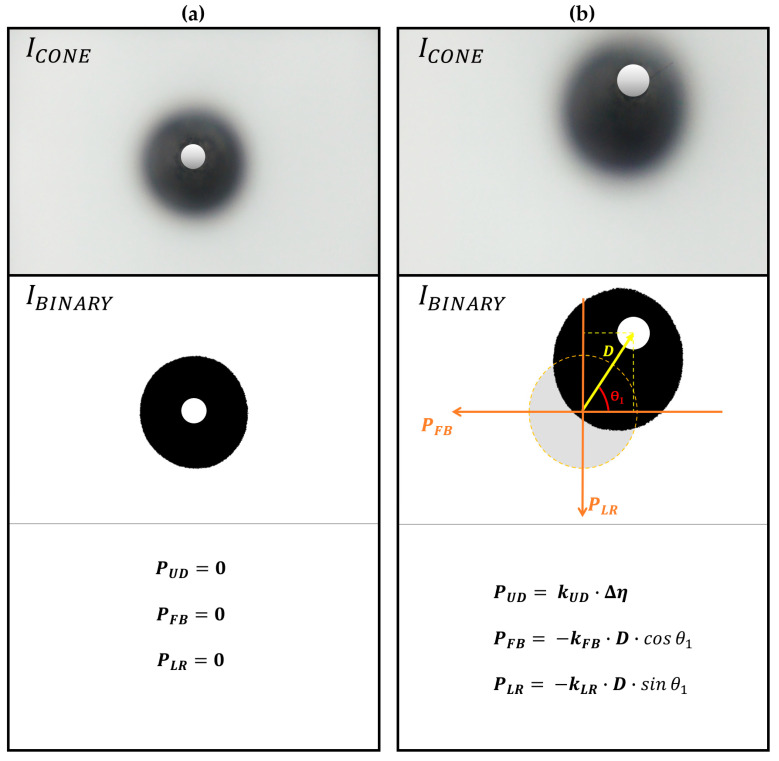
An example of how to measure the deviation distance ***D*** and compute each pressure component, including $P_{UD}$, $P_{FB}$, and $P_{LR},$ in a situation where a combined pressure is applied to the fingertip surface. (**a**) $I_{CONE}$ and its corresponding $I_{BINARY}$ in the absence of pressure (Case: $P_{UD}=0,{\;P}_{LR}=0,{\;and\;P}_{FB}=0)$. (**b**) $I_{CONE}$ and its corresponding $I_{BINARY}$ under a complex pressure with *θ*_1_ = 45° (Case: $P_{UD}\neq 0,$ $P_{S}=\sqrt{P_{FB}^{2}+P_{LR}^{2}}$).

Figure 7 and Figure 8 provide an illustrative depiction of how the proposed pressure sensor functions as an omnidirectional pressure detector when a camera-captured cone image ${(I}_{CONE})$ is provided.

For example, Figure 7a shows how $I_{CONE}$ varies with increasing vertical pressure applied to the fingertip.

The diameter (Φ) of the DF cone increases in correspondence with the increment in pressure.

Actually, the measurement of Φ was determined through the delta black pixel (Δη), which is defined as the alteration in the number of black pixels present within the binary image $I_{BINARY}$ as against the initial state. The binary image is derived from thresholding the original image $I_{CONE}$.

Thresholding is a process in which a specific pixel intensity value is chosen as a threshold. Pixels with intensities below this threshold are designated as black, while those with intensities above the threshold are designated as white. This operation transforms the image $I_{CONE}$ into its corresponding binary image $I_{BINARY}$, focusing on the regions of interest while disregarding irrelevant parts.

Because now there are only two types of pixels (black and white) on the binary image $I_{BINARY}$, counting the black pixels gives a direct measure of the number of black pixels on the DF cone, which is directly proportional to the diameter (Φ) of the DF cone in the camera-captured image.

This relationship provides a way to estimate the changes in pressure applied to the fingertip surface based on the observed changes in the cone’s diameter in the image.

Importantly, it should be noted here that the delta black pixel (∆η) serves as a quantifiable measure of the changes (ΔΦ) in the DF cone’s diameter (Φ) due to the applied pressure on the fingertip.

The change in black pixel count in the binary image $I_{BINARY}$ corresponds to the variations in the size of the DF cone captured by the camera and is directly related to the pressure exerted on the fingertip surface.

The main reason to use the delta black pixel (∆η) as a measurement is because it is suitable for avoiding potential issues caused by oblique camera angles in that the number of black pixels proportionally changes when there is a change in the DF cone’s diameter (Φ).

When the camera captures the DF cone from an oblique angle, especially under lateral pressure applied to the fingertip, the resulting image might not accurately represent the circular shape of the cone.

This distortion can lead to inaccuracies if you directly measure the cone diameter from the image.

By relying on the change in the number of black pixels, which corresponds to the variations in cone diameter, you can bypass distortions caused by skewed angles and obtain a more accurate representation of the applied pressure. This method helps maintain the reliability and accuracy of the pressure measurement even in the presence of side pressures.

Figure 7b illustrates an experimental outcome demonstrating the measurement of pressure applied from the left side of the fingertip.

As pressure is increased, the vertex of the DF cone deviates further from the center of the camera’s field of view (FOV).

To quantify this deviation, the distance (*D*) between the vertex of the DF cone and the center of the camera FOV is measured in terms of straight-line distance in pixels. This deviation distance (*D*) serves as a measure of the side pressure applied to the fingertip. The increase in *D* reflects the increasing lateral pressure on the fingertip.

Figure 8 provides an illustrative example of a scenario where a combined pressure is applied. This combined pressure involves both upward and side pressures exerted on the fingertip surface.

The deviation distance *D* is directly measured by determining the straight-line distance in pixels between the vertex of the DF cone and the center of the camera’s field of view (FOV).

With *D* known, it becomes possible to calculate the individual pressure components, including $P_{UD}$ (up–down pressure), $P_{FB}$ (forward–backward pressure), and $P_{LR}$ (left–right pressure), using the Equation (2).

(2) $$P_{UD}=k_{UD}\cdot \Delta \eta ,\;P_{FB}=-k_{FB}\cdot D\cdot cos\theta_{1},\;\mathrm{and}\;P_{LR}={-k}_{LR}\cdot D\cdot sin{\;\theta }_{1},$$

where $k_{UD,\;}\;k_{FB},and\;k_{LR\;}$ are the proportional constants of each pressure component (see Section 3.4 for the constants).

### 3.4. Linearity Comparison between DFP, OFP, and FSR Sensors

In this context, we conducted a comparison of linearity among three pressure sensors: the DFP sensor, the OFP sensor [55], and the commercially available FSR sensor [56].

**Figure 9:**
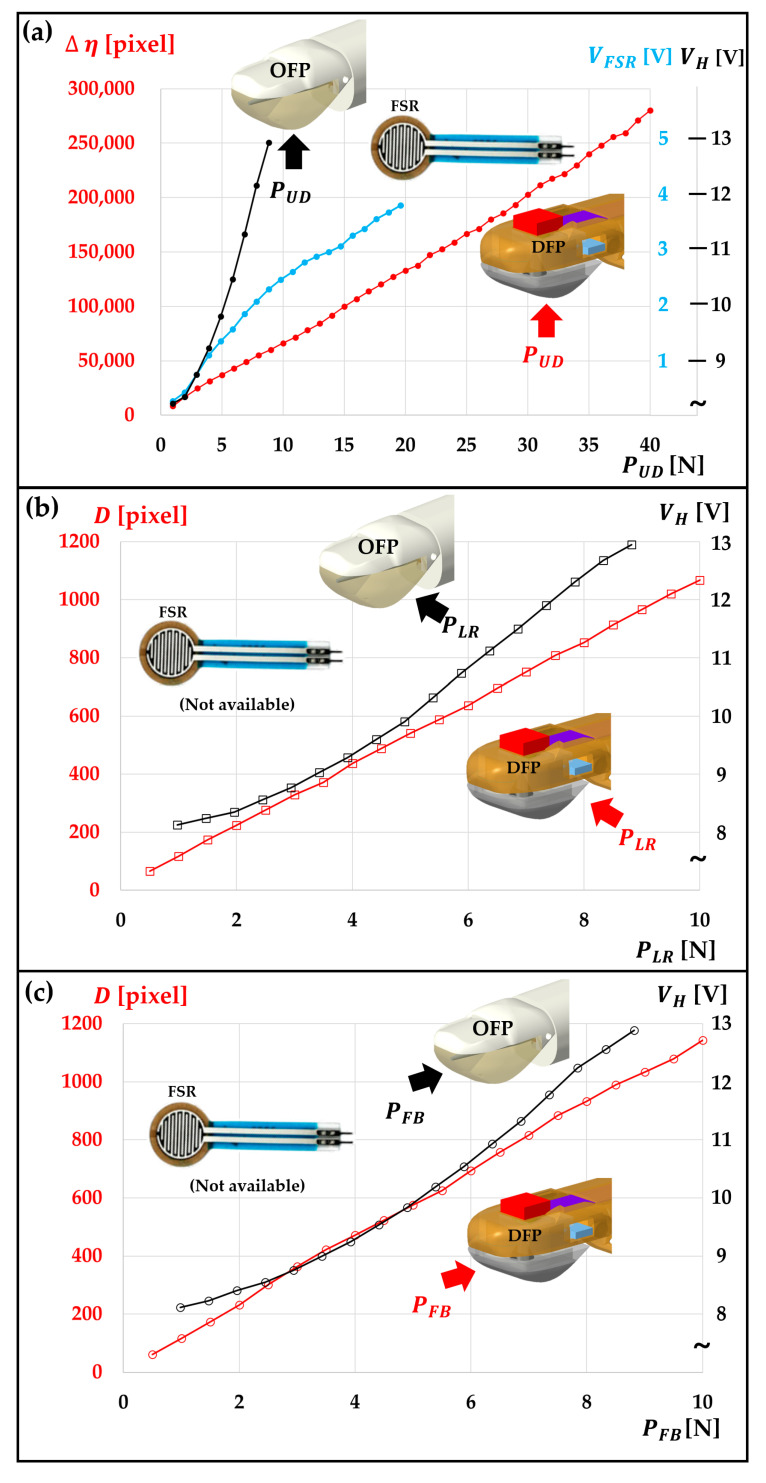
Comparison of three sensors’ response in terms of their working range and linearity across different pressure levels in which the sensor response metrics can be either Δη (DFP), *D* (DFP), V_H_ (OFP). or V_FSR_ (FSR), depending on the sensor being used. (**a**) Sensor response versus pressure under upward pressure conditions. (**b**) Sensor response versus pressure under left–right pressure conditions. (**c**) Sensor response versus pressure under front–back pressure conditions.

To do this, we measured the response of each sensor by applying pressure within the range of 0 to 40 N, while varying the pressed positions as depicted in Figure 9. We have plotted the responses of the three sensors against the applied pressure, facilitating a straightforward comparison of their linearity and operational range.

Figure 9a displays the measurement results of the upward pressure against the fingertip, whereas Figure 9b,c shows the cases when there is a side pressure, each for $P_{LR}\;{and\;P}_{FB}.$

An examination of the sensor response illustrated in Figure 9 indicates that our DFP sensor stands out not only for its exceptional omnidirectionality but also for its superior linear correlation between sensor response and pressure across all pressure directions.

This remarkable performance can be largely attributed to the utilization of the delta black pixel algorithm and the membrane configuration of DEMW-ⅠⅠ. The former is capable of accurately calculating the diameter of the DF cone, the latter helps substantially extend the elastic dynamic range of the membrane wall.

While the FSR402 sensor demonstrates favorable sensitivity and a wide measurable pressure range, its precision falls short. This issue of limited precision has been identified as the primary drawback of FSR402 sensors in various preceding research works [59,60,61]. Furthermore, the FSR402 exhibits subpar linearity and lacks the ability to offer omnidirectional sensitivity.

Conversely, the OFP sensor exhibits omnidirectional responsiveness across a broad pressure range, with a high level of sensitivity [55].

Nonetheless, it possesses significant drawbacks, including a relatively narrow operational range, especially when exposed to upward pressure, and a comparatively high lower LOD of side pressure. Consequently, it falls behind when compared to the DFP sensor.

**Figure 10:**
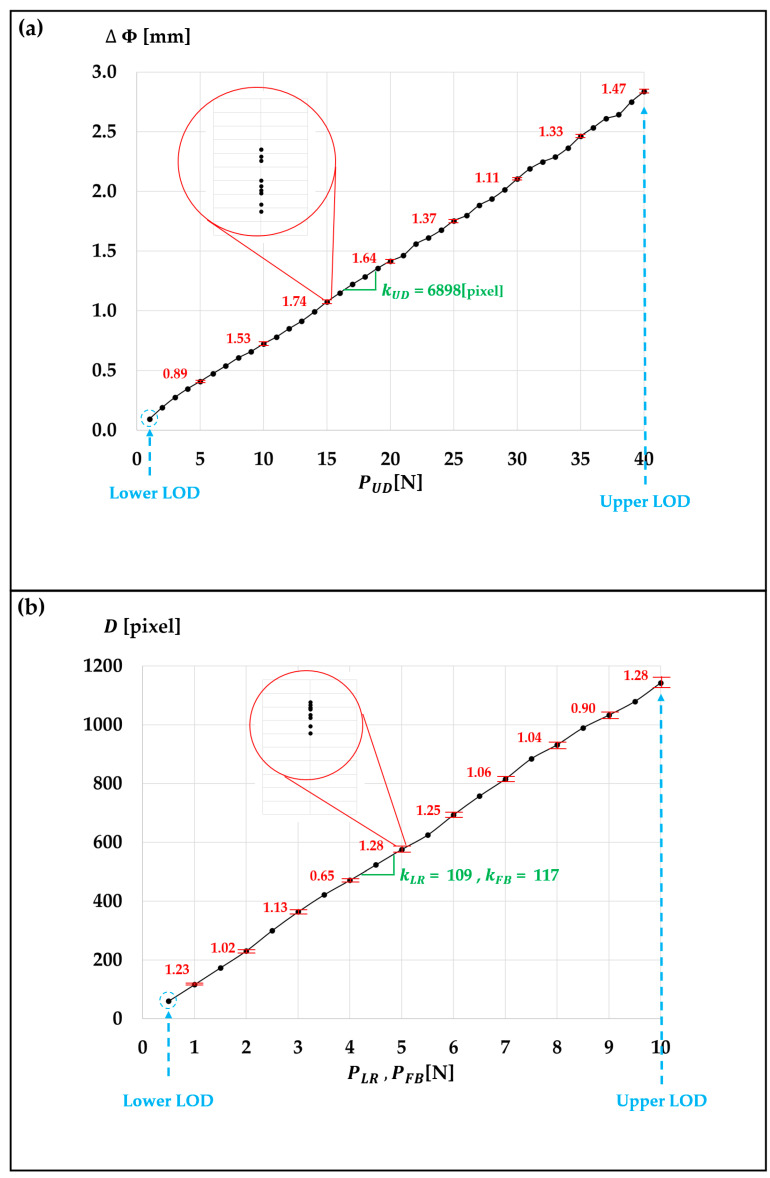
Assessment of the DFP sensor’s stability carried out through an examination of the relative standard deviation for each pressure level across ten separate trials. In the graphical representation, the error bars reflect the relative standard deviation at specific pressure levels, and the proportional constants corresponding to each pressure component, such as $k_{UD\;},{\;k}_{LR}{,\;and\;k}_{FB}$, are also indicated on the graph. (**a**) Plot of ΔΦ versus $P_{UD}$ and (**b**) plot of *D* versus $P_{S}$.

Furthermore, we assessed the reliability of the DFP sensor, as depicted in Figure 10. In this evaluation, ten measurements were performed for each pressure level, covering the range from the lower limit of detection to the upper limit of detection with an increment of 0.5 N or 1 N in pressure level.

Analyzing the above-collected data, it becomes evident that the sensor response is almost directly proportional to the applied pressure with a relatively low standard deviation.

This outcome clearly indicates that the DFP sensor exhibits a robust linear correlation between pressure level and sensor response, irrespective of the direction of pressure applied to the fingertip.

Consequently, we can deduce that our pressure sensor has sufficient omnidirectionality while maintaining the linear measurement characteristics of both ΔΦ and *D* with respect to pressure across all pressure directions.

## 4. Conclusions and Discussion

In this study, a Dynamic Focusing Pressure (DFP) sensor, which is a DF cone-based omnidirectional fingertip pressure sensor, has been proposed.

As presented in the provided information, it offers a remarkable advancement in the field of robotic fingertip pressure sensing. The sensor’s design combines innovative approaches to achieve exceptional omnidirectional sensitivity, broad working range, accuracy, and linearity in pressure measurements. Such noteworthy advantages are mainly attributed to three factors including the following:

(1) Delta black pixel algorithm: This offers a precise and reliable means of measuring the diameter of the DF cone captured by the camera sensor. By focusing on the change in the number of black pixels within the cone’s image, the algorithm provides a direct correlation to the cone’s size, which in turn corresponds to the applied pressure on the fingertip. The delta black pixel algorithm addresses potential challenges arising from oblique camera angles. It mitigates distortions caused by skewed perspectives, ensuring that accurate measurements are obtained even when the camera captures the DF cone from non-optimal angles. This characteristic enhances the sensor’s accuracy and reliability in diverse scenarios, contributing to its robustness across different pressure directions.

(2) Double-floored elastic membrane wall (DEMW): The incorporation of DEMW within the DFP sensor design expands the sensor’s elastic dynamic range. This double-floored membrane structure, composed of elastomeric materials with different hardness levels, allows for enhanced sensitivity and an extended working range compared to conventional single-layer membrane walls. This feature opens up a high potential for the DFP sensor’s application in the field of robotic finger pressure sensing, where accurate measurements in a wide operational range of pressure levels are crucial.

(3) Dynamic focusing (DF) cone: The utilization of a DF cone in the DFP sensor offers several noteworthy advantages. First of all, the DF cone’s mechanism enables the sensor to accurately and quite readily capture pressure-induced changes from the cone’s diameter. This unique feature enhances the sensor’s sensitivity and allows for the calculation of pressure variations with high precision. Moreover, the DF cone contributes to the DFP sensor’s exceptional omnidirectional sensitivity and makes the DFP sensor’s design straightforward and cost-effective. By accommodating pressure changes from various angles and directions, the DF cone facilitates the sensor’s ability to provide reliable pressure measurements across various pressure directions.

Comparative assessments against other pressure sensors further highlight the DFP sensor’s advantages. It offers remarkable sensitivity (up to 0.07 mm/N), a broad working range (up to 40 N), and a straightforward fabrication process.

The evaluation of the DFP sensor’s stability revealed promising results. Through meticulous analysis of relative standard deviations across ten trials for each pressure level, we observed that the sensor response exhibited a direct proportionality to the applied pressure. The narrow range of relative standard deviation values of less than two attests to the consistency and reliability of the DFP sensor’s performance under various pressure conditions.

The DFP sensor’s consistent linear correlation between pressure level and sensor response, along with low standard deviations, underscores its reliability and suitability for real-world applications.

Nevertheless, our DFP sensor does come with certain limitations. Specifically, when the camera sensor captures the DF cone at an oblique angle, accurately determining the vertex of the DF cone from its skewed image might be challenging.

Accordingly, we needed to manually pinpoint the vertex coordinate because automatic determination often becomes unreliable under such conditions.

Furthermore, the clarity and visibility of the DF cone are susceptible to environmental factors due to placing the DF cone on the inside space of the 3D-printed semi-transparent finger body. Consequently, we needed to maintain consistent illumination conditions and an experimental setup throughout the duration of the experiment to mitigate these influences.

Another challenge encountered in the DFP sensor is the relatively constrained operational range of side pressure, which is approximately four times narrower than that of vertical pressure. This limitation stems from the yoke-slit structure’s limited degree of freedom (DOG).

Our ongoing research efforts will be directed towards addressing these limitations.

We aim to develop advanced algorithms that enhance the automatic determination of the vertex coordinate even under oblique angles, ensuring accurate side pressure measurements.

And then we will explore strategies like incorporating an LED (light-emitting diode) to optimize illumination and imaging conditions to minimize the impact of environmental variables on image quality.

Additionally, in our future research endeavors, we are committed to addressing the specific concern related to the operational range of side pressure being narrower than that of vertical pressure. We will focus on innovative design modifications and structural enhancements aimed at expanding the degree of freedom (DOG) within the yoke-slit structure. By strategically improving this aspect, we aim to achieve a more balanced operational range for both side and vertical pressures, thus enhancing the overall capabilities and versatility of the DFP sensor.

We anticipate that the DFP sensor’s capabilities hold significant promise for robotic hand applications. Its ability to accurately sense pressure from any direction aligns well with the complex and versatile requirements of robotic manipulation tasks and contributes to improving the dexterity and efficiency of robotic hand applications across various industries.

## Figures and Tables

**Table 1:** Comparison of performance between the proposed DFP sensor and the previously reported sensors.

**Sensing Mechanism**	**Technology**	**Sensing Direction**	**Sensing Range [N]**	**Sensitivity** **(Resolution)**	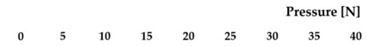
Insight [48]	Camera with soft elastomer	Uni-directional	0~2 N	10 mm/N (Res = 0.03N)	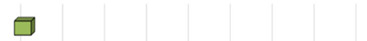
ReSkin [49]	Magnetic field	Uni-directional	0~2.5 N	2.7 mm/N (Res = 0.2 N)	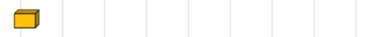
Lancaster [50]	Time-of-flight (TOF)	Uni-directional	0~10 N	1.3 mm/N (Res = N/A)	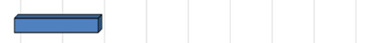
BioTac [51,52]	Fluid-based sensing	Uni-directional	0~10 N	0.34 mm/N (Res = 0.26N)	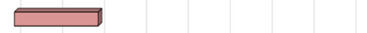
Gelsight-based [53,54]	Camera with soft elastomer	Uni-directional	0~22 N	0.2 mm/N (Res = 1.9 N)	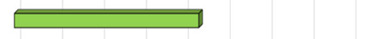
OFP Sensor [55]	Hall effect sensing	Omnidirectional	0.4~9 N	613.4 mm/N (Res = N/A)	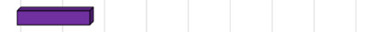
FSR 402 [42,56]	Resistive sensing	Uni-directional	2~20 N	328 mm/N (Res = N/A)	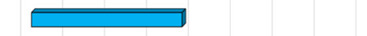
DFP Sensor [this work]	▪ Dynamic Focusing Cone ▪ DEMW-II (See Section 3.2)	Omnidirectional	0~40 N *	0.07 mm/N (Res. = See Figure 6)	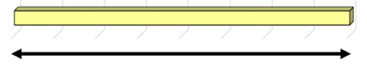
					Typical human touch pressure range [57,58]

* Measured on the condition that *P_UD_* ≠ 0, *P_S_* = 0.
